# Fatigue Resistance of Dissected Lower First Molars Restored with Direct Fiber-Reinforced Bridges—An In Vitro Pilot Study

**DOI:** 10.3390/polym15061343

**Published:** 2023-03-08

**Authors:** Veronika T. Szabó, Balázs Szabó, Noémi Barcsayné-Tátrai, Csongor Mészáros, Gábor Braunitzer, Balázs P. Szabó, Lippo Lassila, Sufyan Garoushi, Márk Fráter

**Affiliations:** 1 Department of Operative and Esthetic Dentistry, Faculty of Dentistry, University of Szeged, H-6720 Szeged, Hungary; 2 Department of Periodontology, Faculty of Dentistry, University of Szeged, H-6720 Szeged, Hungary; 3 DicomLAB Dental Ltd., H-6726 Szeged, Hungary; 4 Department of Food Engineering, Faculty of Engineering, University of Szeged, H-6725 Szeged, Hungary; 5 Department of Biomaterials Science and Turku Clinical Biomaterials Center—TCBC, Institute of Dentistry, University of Turku, FI-20520 Turku, Finland

**Keywords:** molar dissection, short fiber-reinforced composite, inlay-retained bridge, fatigue resistance, periodontal support, furcation involvement

## Abstract

The aim of this research was to evaluate the mechanical impact of utilizing different fiber-reinforced composite (FRC) systems to reinforce inlay-retained bridges in dissected lower molars with different levels of periodontal support. A total of 24 lower first molars and 24 lower second premolars were included in this study. The distal canal of all molars received endodontic treatment. After root canal treatment, the teeth were dissected, and only the distal halves were kept. Standardized class II occluso-distal (OD) (premolars) and mesio-occlusal (MO) (dissected molars) cavities were prepared in all teeth, and premolar–molar units were created. The units were randomly distributed among four groups (n = six/group). With the aid of a transparent silicone index, direct inlay-retained composite bridges were fabricated. In Groups 1 and 2, both discontinuous (everX Flow) and continuous (everStick C&B) fibers were used for reinforcement, while in Groups 3 and 4, only discontinuous fibers (everX Flow) were used. The restored units were embedded in methacrylate resin, simulating either physiological periodontal conditions or furcation involvement. Subsequently, all units underwent fatigue survival testing in a cyclic loading machine until fracture, or a total of 40,000 cycles. Kaplan–Meyer survival analyses were conducted, followed by pairwise log-rank post hoc comparisons. Fracture patterns were evaluated visually and with scanning electron microscopy. In terms of survival, Group 2 performed significantly better than Groups 3 and 4 (*p* < 0.05), while there was no significant difference between the other groups. In the case of impaired periodontal support, a combination of both continuous and discontinuous short FRC systems increased the fatigue resistance of direct inlay-retained composite bridges compared to bridges that only contained short fibers. Such a difference was not found in the case of sound periodontal support between the two different bridges.

## 1. Introduction

In the past few decades, the popularity and availability of implant-retained restorations has escalated. However, the number of complications (peri-implantitis and peri-implant mucositis) associated with their use has also increased [1,2]. It is also known that the success rates of implants in periodontal patients lag behind the success rates seen in periodontally unaffected patients [3]. Due to the above mentioned reasons, patients still prefer to keep their own dentition. Thanks to modern approaches and armamentarium of restorative dentistry, it is now possible to conservatively treat many situations that would once have inevitably led to extraction [4]. Resective surgical approaches, such as dissection, are still worth considering as clinically relevant treatment options versus extraction and implant placement [5,6]. The dissection of a lower molar tooth, for either endodontic or periodontal reasons, aiming to preserve part of it after the removal of the non-savable part is an intervention that serves such a purpose. According to DeSanctis and colleagues, endodontic indications include endodontic failure (perforation, file separation, etc.), root caries, resorption, and fracture [7]. In such cases, the alveolar bone around the remaining root is usually predominantly intact. According to the findings of Carnevale and colleagues, periodontal indications can be severe furcation involvement, severe bone loss simultaneously affecting one or more roots, and severe recession or fenestration [8]. In these cases, the amount of alveolar bone around the retained root significantly affects the material choice and the type of restoration [9]. Some root resection studies with 5–10 years of follow-up showed success rates of 62–100%, which is quite promising in such a demanding situation [10]. When dissection is performed on a lower molar tooth, in most cases, the restoration is extended toward the direction of the removed root, possibly involving the neighboring one also. Since on many occasions the neighboring tooth is intact (or contains only a minor conservative restoration), restoring the edentulous span with an intracoronally retained bridge could be an ideal solution for such situations, following the principle of minimally invasive dentistry. Minimally invasive FRC bridges appear to be a good choice for restoring the affected edentulous span, as they are considered viable medium-term management alternatives for replacing single anterior or posterior teeth to traditional porcelain-fused-to-metal bridges [11]. An obvious and also widely used approach is to prepare an indirect restoration after digital or conventional impression taking. However, there is an increasing amount of evidence to support the use of fiber-reinforced direct restorations for more extensive defects [12]. Such restorations offer the advantages of minimally invasive preparations, lower costs, faster workflows, and better repairability. Furthermore, the mechanical, aesthetic, and handling properties of dental composites have shown a rapid development over the past few decades [13]. Since the development of pre-impregnated fiber bundles more than 20 years ago, the reinforcement of composites has gained increasing interest [14,15]. So far, limited information is available on their longevity and clinical behavior, but the available clinical studies of fiber-reinforced composite (FRC) fixed partial dentures demonstrate a high overall survival with predictable performance outcomes when used as a medium-term management alternative for replacing single anterior or posterior teeth [11]. The five-year survival rates of FRC bridges range between 73 and 93% [16,17].

Traditionally, continuous fibers are used for the reinforcement of direct inlay-retained composite bridges. Studies so far have focused on the type of long fibers incorporated in the bridge [18,19], the different framework designs with different fiber orientation [20,21], and the effect of the material and the occlusal morphology of the pontic [22]. The purpose of unidirectional long fibers is to improve the mechanical properties of the direct composite bridges. Whenever the clinical situation enables the usage of unidirectional long fibers, they are the best to achieve reinforcement within the structure of the restoration. This is due to the fact that unidirectional fibers give anisotropic mechanical properties to the composite and are known to be suitable for applications where the highest stress occurs. Reinforcing efficiency, evaluated by the Krenchel’s factor, of unidirectional fibers is theoretically 100%, which means that reinforcing properties can be achieved and concentrated in one distinctive direction.

However, discontinuous, short fiber-reinforced composites (SFRCs) have been utilized for various other restorative purposes lately [23,24,25,26]. SFRC materials hold the promise of crack-arresting within the SFRC structure due to specific, unique features of the material, such as aspect ratio, critical fiber length, fiber loading, fiber orientation, and adhesion between the matrix and the fiber [27,28]. This is actually measurable in the remarkable fracture toughness values of these materials. Fracture toughness is a property of the material that describes the resistance of brittle materials to the catastrophic propagation of flaws under loading, which therefore shows the damage tolerance of the material [27]. This means that a material with a high fracture toughness is able to resist crack initiation and propagation. Since the fibers are orientated randomly in SFRCs, the mechanical properties as well as the ability for potential reinforcement are equal in all directions and are three-dimensionally isotropic [29]. The features/characteristics of these two distinctive fibers are listed in Table 1. According to the manufacturer’s instructions, fibers should be covered with particulate-filled composites (PFCs) from all directions to avoid moisture uptake from the oral cavity. Yet, more and more studies show that the fracture and fatigue resistance of a restoration can be improved when the flowable SFRC is not covered with PFC [30,31,32,33]. Thus, the question arises whether SFRCs could be utilized to improve an FRC bridge. To the best of our knowledge, no one has yet tested the flowable SFRC in combination with long fibers to fabricate a direct inlay-retained bridge for restorative purposes. Furthermore, different bone levels have not been simulated in such a restorative situation at all. These give novelty to the proposed study design.

We aimed to investigate whether continuous or short and discontinuous fibers are better to reinforce inlay-retained direct bridges in terms of fatigue resistance and fracture patterns. We also sought to investigate how periodontal support may influence this.

The null hypotheses were that there would be no difference between the test groups either in (1) fatigue resistance or (2) in the fracture patterns.

## 2. Materials and Methods

### 2.1. Sample Selection

All procedures of the study were approved by the Ethics Committee of the University of Szeged (approval no. 4029), and the study was designed in accordance with the Declaration of Helsinki.

In our study, 24 mandibular second premolars and 24 mandibular first molars, previously extracted due to periodontal or orthodontic reasons, were selected and included. All teeth were used within 6 months of extraction. Soft tissue residues, cementum, and calculus covering the root surfaces were removed with hand and ultrasonic scalers. Until they were used, the teeth were stored in 0.9% saline solution at room temperature.

The primary inclusion criteria were the visual absence of caries or root cracks, previous endodontic treatment, posts or crown, or resorptions. Regarding the coronal dimensions of the molar teeth, approximately 90% of the specimens ranged from 10 to 10.9 mm in the bucco-lingual dimension, and the rest were between 11 and 12 mm. The mesio-distal dimension of the specimens was also measured; a mean was calculated and specimens that fell within the ±10% range of the mean were included. The height of the specimens was between 8 and 9 mm measured from the cementoenamel junction (CEJ). The length of the distal roots fell in the 14–16 mm range. Regarding the coronal dimensions of the premolars, 90% of the teeth ranged between 9 and 10 mm in the bucco-lingual dimension. As for the mesio-distal dimension, 90% of the samples measured between 7 and 7.5 mm [24,30].

### 2.2. Sample Preparation

All procedures were performed by the same trained operator. The lower first molars were dissected with a vertical cut through the oro-vestibular bisector of the crown toward the fornix, and only the distal tooth halves were kept for further procedures.

The sectioned surfaces were smoothened to have a cleansable non-retentive surface. In the hemisected distal tooth halves, the pulp chambers were deroofed and endodontic treatment of the distal canal(s) was performed according to our previous protocol [34]. The root canals were instrumented with Pathfiles (1-2-3) and ProTaper (S1-S2-F1-F2-F3) (Dentsply Maillefer, Ballaigues, Switzerland) to the working length. The specimens were irrigated with 5% NaOCl alternating with 10% EDTA (ethylenediaminetetraacetic acid) with a 2 mL syringe and 25-gauge needle. Root canal filling was performed by matched-single-cone obturation with a master cone matching the final instrument used for preparation and sealer (AH plus; Dentsply Maillefer).

In the hemisected distal tooth halves, standardized mesio-occlusal (MO) in all premolars occluso-distal (OD) cavities was prepared according to Cara et al. [35].

All specimens received the same adhesive treatment. The pulp chambers of the dissected molars and the proximal boxes of the premolars were roughened with a carbide bur, cleaned, and after selective enamel etching for 15 sec with 37% orthophosphoric acid, a one-step self-etch adhesive system was applied (G-Premio Bond, GC Europe, Leuven, Belgium) according to manufacturer’s instructions. The adhesive was light-cured for 40 s using an Optilux 501 quartz–tungsten–halogen light-curing unit (Kerr Corp., Orange, CA, USA). The average power density of the light source, measured with a digital radiometer (Jetlite light tester; J. Morita USA Inc., Irvine, CA, USA) before the bonding procedure, was 840 ± 26.8 mW/cm^2^. After photopolymerization, the proximal boxes and the pulp chambers were filled up to the level of the occluso-pulpal wall with a composite filling material (G-aenial Posterior A3, GC Europe) and light-cured for 20 s.

### 2.3. Direct Bridge Fabrication

**Figure 1:**
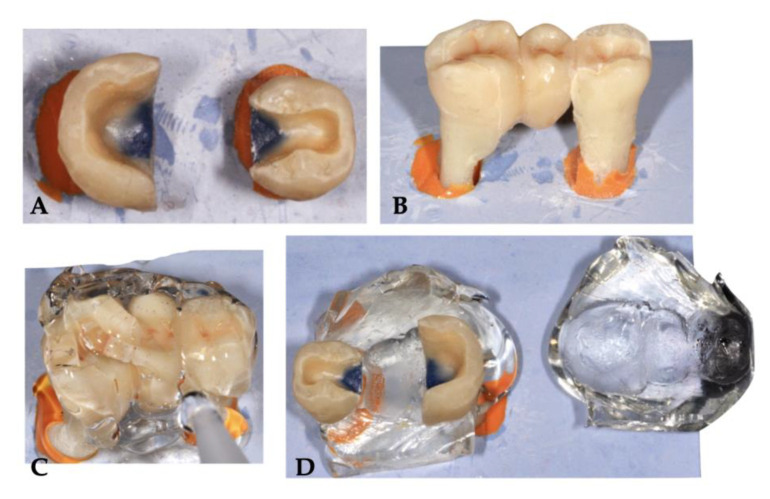
Steps of fabricating a standardized, transparent silicone index to help the fabrication of the future direct, inlay-retained bridges.

The premolars and dissected molars were paired, forming units, and fixed in a gypsum block using light body silicone impression material. Silicone was an ideal solution for fixing, as the roots could be completely and easily removed from it at any time during the procedures, aiding handling of the samples (Figure 1A). In order to standardize the size and the shape of the direct, inlay-retained bridges, a transparent silicone index was individually made for each sample in the following way: a laboratory-technician-made prefabricated, inlay-retained composite bridge was placed into the cavities of each unit (Figure 1B), all potential gaps were blocked out with a temporary filling material, and a template was made from a transparent silicone impression material (Exaclear, GC Europe) to obtain a silicone index of the future restoration (Figure 1C). After setting, the silicone index was sectioned horizontally at the equator with a scalpel and the bridge was removed (Figure 1D).

During the fabrication of the inlay-retained bridges different FRC were applied (Table 1) with different designs (the utilized materials and designs are summarized in Table 2).

**Figure 2:**
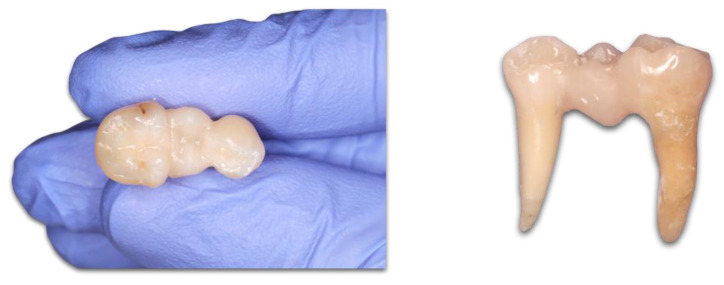
The standardized direct FRC bridges were finished and polished.

In Groups 1 and 2, after the adhesive treatment of the cavities (see above), a flowable SFRC material (everX Flow dentin shade, GC Europe) was molded to the horizontally cut silicone index to create the gingival part of the pontic to the level of the occlusal boxes of the teeth and was then photopolymerized for 40 s. Then, a flowable SFRC was applied to the walls of the prepared cavities covering the cervical and one third of the box. After this, a bundle of long, unidirectional FRC (everStick C&B, GC Europe) was cut at the appropriate length, placed in position, and photopolymerized. The remaining occlusal, two thirds of the bridge, was molded using a flowable SFRC again, and photopolymerization (40 s) of the last occlusal layer was performed through the transparent silicone index to standardize the occlusal anatomy. The restorations were finished with fine granular diamond burs (FG 7406-018, Jet Diamonds, USA and FG 249-F012, Horico, Germany) and aluminum oxide polishers (OneGloss PS Midi, Shofu Dental GmbH, Ratingen, Germany) (Figure 2).

In Groups 3 and 4, the direct composite bridges were made of only a flowable SFRC (everX Flow dentin shade). The clear silicone indexes were created in the aforementioned way, but instead of the horizontal cut, only 3 holes were punched in them through which the tip of the flowable SFRC was inserted, and the bridge was molded. After the adhesive treatment of the cavities (see above), the negative space encapsulated by the clear silicone index was molded incrementally by the flowable SFRC. Photopolymerization was made through the transparent silicone index. Each layer was light-cured for 40 s. The restorations were finished and polished the same way as in Group 1 and 2.

### 2.4. Embedding of the Samples

All units restored in the aforementioned way were embedded in a special methacrylate resin (Technovit 4004, Heraeus-Kulzer, Wertheim, Germany) in the following way: samples of Groups 1 and 3 were embedded in accordance with intact periodontal conditions 2 mm from the CEJ, while in Group 2 and 4, embedding was performed and the level of embedding was set at 6 mm apical to the CEJ, simulating furcation involvement [34,36]. The simulation of periodontal ligaments was achieved with a latex separating liquid (Rubber-Sep, Kerr, Orange, CA, USA), which was applied in one layer to the roots according to the level of the planned embedding, before embedding [34,37].

**Figure 3:**
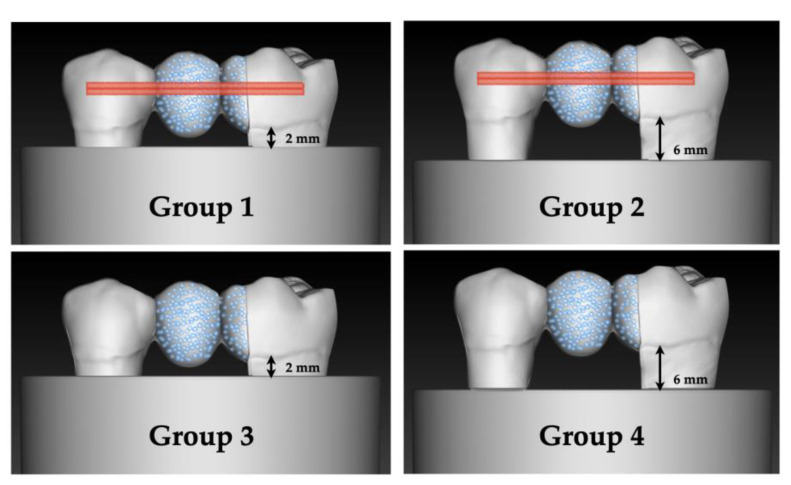
Schematic pictures of the 4 study groups.

The 4 study groups with the corresponding levels of embedding are shown in Figure 3.

### 2.5. Mechanical Testing

**Figure 4:**
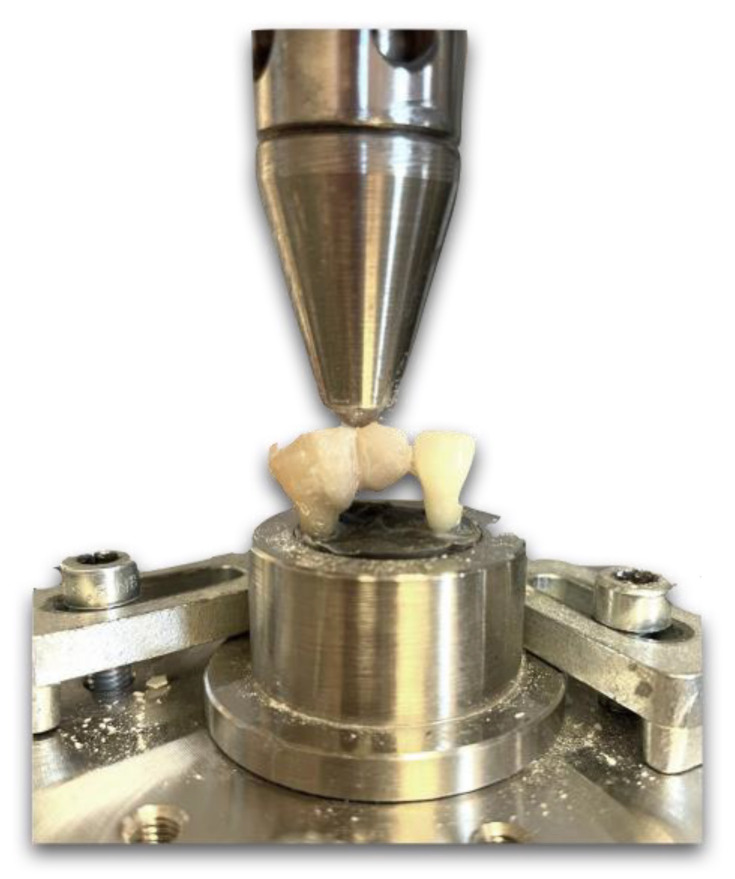
During the accelerated fatigue test, the load was applied at the connector part of the restored units with a 5 mm wide, round-ended metallic tip.

The samples were subjected to an accelerated fatigue-testing protocol [18], performed with a hydrodynamic testing machine (Instron ElektroPlus E3000, Norwood, MA, USA). Cyclic isometric loading was applied at the connector part of the restored units with a 5 mm wide, round-ended metallic tip (Figure 4). During the preloading, the samples had to endure a cyclic load at a frequency of 5 Hz. First, the load was continuously increased to 100 N in 5 s, then dynamic loading was applied with a force of 100 N for 5000 cycles. The load was then increased by 100 N each time up to 800 N, and 5000 cycles were completed for each 100 N increase. The teeth were loaded until fracture or until the total number of cycles was reached (40,000 cycles).

After the loading test, the fractures of all fractured specimens were observed visually and were classified into two categories according to the extent of the fracture line. A fracture was considered favorable (reparable) if the composite restoration fractured with or without the tooth structure coronally to the simulated bone level, but not below it. Fractures reaching below this level were considered unfavorable (irreparable).

## 3. Results

**Figure 5:**
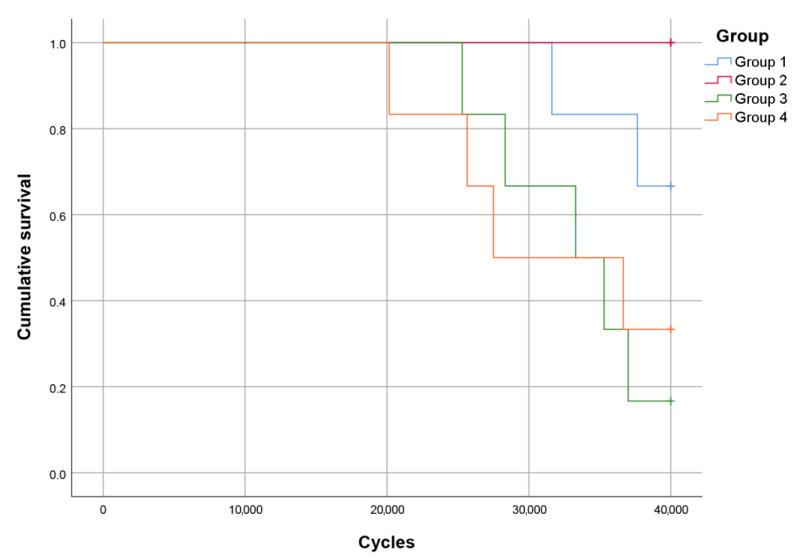
Kaplan–Meier survival curves.

The Kaplan–Meier survival curves of the tested groups are presented in Figure 5, and the results of the post hoc log-rank pairwise comparisons (*Mantel–Cox*) are given in Table 3.

The fatigue resistance of Group 2 was significantly superior to Group 3 (*p* = 0.004) and Group 4 (*p* = 0.018), but the rest of the comparisons did not indicate a significant difference. Consequently, the first null hypothesis was rejected.

In terms of the fracture patterns (Table 4), most of the samples in Groups 1 and 2 survived, while Groups 3 and 4 were characterized by an almost equal number of favorable and unfavorable fractures. Therefore, the second null hypothesis regarding fracture patterns was rejected too.

## 4. Discussion

The present study focused on the possible effects of FRC system choice and the remaining bone level on the fatigue resistance of lower dissected molar teeth that were restored with direct, inlay-retained FRC bridges. As in the recent studies carried out by the authors, cyclic loading was used instead of static load-to-fracture testing in this study [23,30,31]. When testing the tooth-restoration units, cycling loading is considered to be more suitable to model oral clinical conditions than static testing, as during cyclic loading repetitive forces are generated, which is closer to the conditions of chewing [31]. Additionally, as pointed out by Le Bell-Rönnlöf, fatigue more often leads to the fracture of a restored tooth than static forces [38]. Accelerated fatigue was introduced as a rational middle ground between the load-to-fracture test and other, more sophisticated and time-consuming fatigue tests [39], and it has been used in several studies since its introduction [40,41,42].

In this study, utilizing a combination of continuous glass fiber bundles and discontinuous, short fibers in the direct composite bridge (Group 2) significantly increased the fatigue resistance of the restored premolar–molar units compared to the groups utilizing only SFRCs as the reinforcement within the bridge (Group 3, *p* = 0.004 and Group 4, *p* = 0.018). Therefore, our first null hypothesis was rejected. The reason behind our initial results could be the unique structure of the used continuous fibers, along with the SFRCs. The everStick C&B (GC Europe) used in this research contains unidirectional pre-impregnated E-glass fiber bundles embedded in a PMMA (polymethyl methacrylate)/bis-GMA (bisphenol glycidyl dimethacrylate) matrix. Long, continuous, unidirectional E-glass fibers have an anisotropic property, meaning that they provide the greatest reinforcement against forces perpendicular to the direction of the fibers, which corresponds to the typical axial load in the posterior region [43]. A good impregnation of fibers with the surrounding matrix is important as fiber reinforcement is only successful when the loading force can be transferred from the matrix to the fiber [44]. This is ensured by the bis-GMA/PMMA matrix in case of the utilized everStick C&B. So far, everStick C&B has shown superior results in reinforcing composite bridges in terms of fracture strength compared to non-reinforced direct bridges [44]. Our results are in good agreement with a previous study by Garoushi et al., who evaluated the effect of the FRC substructure on the static load-bearing capacity of particulate filler composites and concluded that supporting particulate filler composites by the FRC substructure increased the load-bearing capacity of the material [45]. This is also in line with the work of Cekic-Nagas et al., who found that the addition of long fibers to the framework significantly affected the load-bearing capacity of FRC restorations compared to the ones that were only using SFRCs for reinforcement [19]. However, they used everX Posterior, which is the paste or packable version of SFRC.

Clinical reports found that debonding of the veneering composite, fiber exposure, and fracture at the pontic or at the connector area are the primary failure types occurring in FRC fixed dental prostheses [46]. To overcome these failures, the framework design and amount of fibers should be increased to improve the rigidity of these bridges [47]. In this pilot study, the flowable version of SFRC (everX Flow) was used with and without continuous fibers to reinforce direct composite bridges. While the packable version of SFRCs contains millimeter-long fibers, the flowable one contains micrometer-long ones [48]. Simultaneously, the aspect ratio is still ideal in the case of the flowable version (between 30 and 94) [48], and the smaller size of the fibers allows a greater volume fraction to be used during the manufacturing of the material compared to the packable version. Due to these features, a flowable SFRC has a little higher fracture toughness compared to the packable version and has also produced slightly better results when restoring major dentinal defects compared to the packable one [23,26]. The anisotropic property of the long fibers and the extreme number of fibers incorporated in the SFRC together might account for the better performance of the combination of short and long fibers compared to short fibers alone. This is in accordance with the findings of Keulemans et al., who evaluated the influence of the framework design on the load-bearing capacity of laboratory-made three-unit inlay-retained FRC bridges [49]. The highest load-bearing capacity was observed with FRC frameworks made of a combination of long, unidirectional FRC and SFRC.

Surprisingly, our results seem to suggest that the amount of periodontal support does not play a crucial role in the fatigue resistance when looking at each tested restorative technique, as the groups within a certain restorative technique with different levels of simulated periodontal support did not differ significantly (comparing Group 1 to 2, and Group 3 to 4). This is in agreement with our earlier findings, where we examined the mechanical behavior of root-amputated intracoronally splinted, periodontally compromised maxillary molars and the presence or absence of furcation involvement did not seem to influence the fatigue survival of the tested specimen [34]. At the same time, these findings contradict those of Soares et al., who found that the amount of bony support influenced the strain that developed in the splinted teeth [50]. It must be noted, however, that Soares et al. examined lower front teeth. Furthermore, Szabó et al. found that furcation involvement as periodontal impairment was a significant factor in weakening root-amputated maxillary molars that were restored by either a direct filling or an overlay [4]. Note that in this study, splinted lower tooth pairs were tested, whereas Szabó et al. tested single upper molars, which might be the reason for the seemingly opposing results. The reason behind our findings might be due to the fact that the inlay-retained FRC bridge not only restores the edentulous spam but simultaneously splints the involved teeth together. Splinting either with FRC or non-FRC restorations has long been the therapy of periodontally compromised teeth. However, our results regarding the influence of periodontal levels must be interpreted with caution as this is so far a pilot study and the amount of samples per group should be increased to strengthen the current findings.

In terms of fracture patterns, irreparable fractures occurred more frequently in the SFRC groups (Groups 3 and 4); thus, our second null hypothesis was also rejected. It should be emphasized that most of the samples in Groups 1 and 2 survived the accelerated fatigue testing cycles. This again shows the importance of combining continuous and discontinuous fibers in the restorative process of direct, inlay-retained bridges. Our results suggest that the amount of periodontal support does not seem to influence the fracture patterns of these bridge units as none of the units in Group 2 (continuous fibers and periodontally compromised situation) fractured during the mechanical testing. This is contrary to our previous findings [34], when upper maxillary, root-amputated teeth were examined, which could account for the different outcomes.

**Figure 6:**
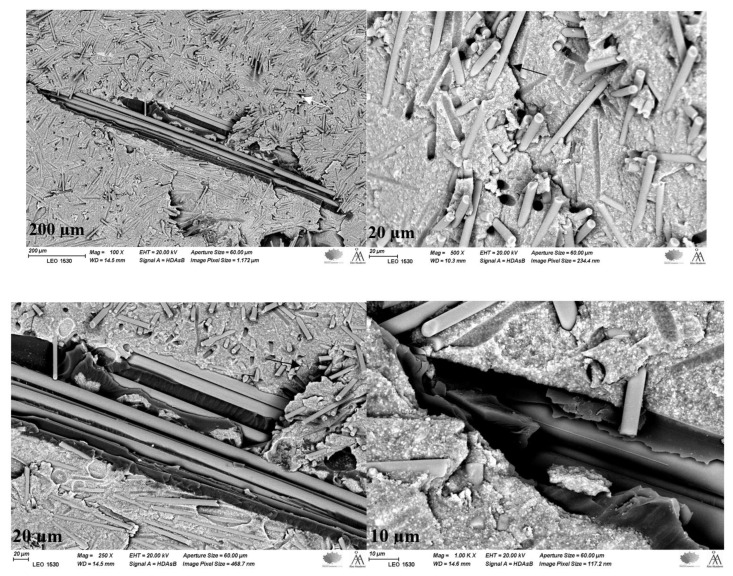
SEM images with different magnifications show how a crack is propagating inside the SFRC material (arrows) to stop when reaching the long fiber bundles in Groups 1 and 2.

A visual analysis revealed that the ways of crack propagation were mostly oblique occlusally gingiva through the connector. SEM images of tested specimens showed that the crack path propagated from the loading surface (occlusally at connector area) to the inner part of composite restoration, where it was stopped/redirected by fibers (Figure 6). It is interesting to note that SFRCs were closely connected to the fiber bundle, which reduced the negative effects of having a weak link between them. Because of the semi-IPN (interpenetrating polymer network) polymer matrix structure, continuous glass fiber bundles (everStick C&B) have a good bonding ability with direct resin composite enabling reliable retained applications. This kind of interlocking or adhesion explains the performance of using both FRC systems together.

On the other hand, Tsujimoto and his colleagues determined that the relationship between mechanical properties and dentine bond durability of SFRCs using universal adhesives showed improvements compared to conventional PFC composites [51]. The ratios of shear fatigue strength and shear bond strength of SFRCs were higher than those of conventional PFC resins. In fact, the superior mechanical properties of SFRCs, especially fracture toughness, could improve the bond durability with adhesives.

We would like to emphasize that in all our tested groups, the external surfaces of the bridges were also made with flowable SFRCs. This was performed in order to maximize the amount of fibers in our restorations. Using flowable SFRCs without PFC coverage is in line with other studies [32,33,52]. According to the findings of Lassila et al., restorations made purely from flowable SFRCs show a significantly higher fracture resistance compared to covered SFRC restorations [51]. As pointed out by Garoushi and colleagues, if the SFRC-core is considered as a crack-stopper, the distance from the surface of the stress initiation point to the SFRC-core is of importance [32].

Finally, the design of this in vitro study has some strengths and limitations. The authors believe that the use of human teeth with similar dimensions, the effort put into standardization of the restored units, and the fact that cyclic loading was applied are obvious strengths. The novelty of the study lies within two features of the study design. Firstly, a flowable SFRC was used without PFC coverage together with unidirectional fibers to potentially strengthen the bridge. Secondly, different periodontal supporting levels have been simulated in this study. Not only that the literature lacks simulations of periodontally compromised situations in mechanical testing, but also, so far there has been no study simulating these severe yet common conditions in the case of inlay-retained direct FRC composite bridges.

The most important limitation is that a relatively low number of specimens were tested as this was a pilot investigation. Furthermore, a bridge with PFC coverage on its external surface should also be tested to see how much of the resistance is attributed to the presence or absence of SFRCs. This is a known limitation of all current in vitro mechanical testing studies, which should be addressed in the future.

## 5. Conclusions

Within the limitations of the study, it can be concluded that in the case of impaired periodontal support, the combination of continuous and discontinuous short glass fibers might increase the fatigue resistance of direct inlay-retained bridges compared to only SFRC-containing bridges. However, when the periodontal support is sound, there seems to be no benefit of adding continuous fibers to the SFRCs in direct composite bridges.

## Figures and Tables

**Table 1:** The investigated fiber-reinforced materials.

Material	Manufacturer	Fiber Orientation	Composition/Fibers Feature *	Mechanical Characteristics *
EverStick C&B	Stick Tech Ltd., GC Group member, Turku, Finland	Unidirectional	Pre-impregnated bundle (1.5 mm) of silanized E-glass fibers (65 vol%) with Bis-GMA, PMMA. ≈ 4000 individual glass fibres (Ø15 μm) per bundle.	FS: 700–800 MPa FM: 15 GPa FT: not available
EverX Flow	GC, Tokyo, Japan	Multi-directional	Bis-EMA, TEGDMA, UDMA, short glass fiber (25 wt%, length 200–300 µm and Ø7 μm), and barium glass.	FS: 146 MPa FM: 9 GPa FT: 2.8 MPa/mm ^0.5^

Bis-GMA, bisphenol-A-glycidyl dimethacrylate; PMMA, polymethylmethacrylate; TEGDMA, triethylene glycol dimethacrylate; UDMA, urethane dimethacrylate; Bis-EMA, ethoxylated bisphenol-A-dimethacrylate; wt%, weight percentage; and vol%, volume percentage. FS, flexural strength; FM, flexural modulus; and FT, fracture toughness. * according to manufacturer data.

**Table 2:** In Groups 1 and 2, both continuous and discontinuous short glass fibers were used, while in Groups 3 and 4 only discontinuous short fibers were used during the restorative procedure.

	Group 1 and 2	Group 3 and 4
Pontic base	EverX flow dentin shade (discontinuous, short fibers)	EverX flow dentin shade (discontinuous, short fibers)
Central glass fibers	EverStick C&B (continuous fibers)	EverX flow dentin shade (discontinuous, short fibers)
	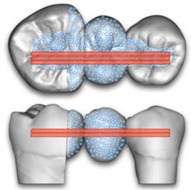	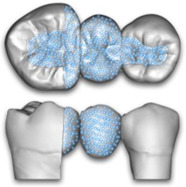
Occlusal anatomy	EverX flow dentin shade (discontinuous, short fibers)	EverX flow dentin shade (discontinuous, short fibers)

**Table 3:** Results of the pairwise comparisons (Mantel–Cox). Asterisk (*) marks significant difference.

Pairwise Comparisons									
	GROUP	Group 1		Group 2		Group 3		Group 4	
		Chi-Square	Sig.	Chi-Square	Sig.	Chi-Square	Sig.	Chi-Square	Sig.
Log Rank (Mantel-Cox)	Group 1			2.195	0.138	3.607	0.058	1.950	0.163
	Group 2	2.195	0.138			8.170	0.004 *	5.568	0.018 *
	Group 3	3.607	0.058	8.170	0.004 *			0.041	0.839
	Group 4	1.950	0.163	5.568	0.018 *	0.041	0.839		

**Table 4:** Fracture patterns.

	Survived	Fractured	Favorable Fracture	Unfavorable Fracture
Group 1	4	2	1	1
Group 2	6	0	-	-
Group 3	1	5	2	3
Group 4	2	4	2	2

## Data Availability

Data are contained within the article.
